# A new strategy and its effect on adherence to intermittent preventive treatment of malaria in pregnancy in Uganda

**DOI:** 10.1186/1471-2393-13-178

**Published:** 2013-09-21

**Authors:** Anthony K Mbonye, Stephanie Yanow, Josephine Birungi, Pascal Magnussen

**Affiliations:** 1 School of Public Health, College of Health Sciences, Makerere University & Commissioner Health Services, Ministry of Health, Box 7272, Kampala, Uganda; 2 Provincial Laboratory for Public Health, University of Alberta, Edmonton, Alberta, Canada; 3 Uganda Virus Research Institute, Entebbe, Uganda; 4 Centre for Medical Parasitology, University of Copenhagen, Copenhagen, Denmark

**Keywords:** Malaria in pregnancy, IPTp, Adherence, Mama kit, Health-Trust Model, Uganda

## Abstract

**Background:**

Few women in Uganda access intermittent preventive treatment of malaria in pregnancy (IPTp) with sulfadoxine-pyrimethamine (SP). Previous studies have shown that high costs, frequent stock-out of drugs, supplies and poor quality of care are the greatest hindrance for women to access health services. In order to increase adherence to IPTp, we conceptualised an intervention that offset delivery care costs through providing a mama kit, created awareness on health benefits of IPTp and built trust between the provider and the client.

**Methods:**

The new strategy was conceived along four constructs namely: 1) ***creating awareness*** by training midwives to explain the benefits of SP and the importance of adhering to the two doses of SP as IPTp to all pregnant women who attended ANC and consented to the study. Midwives were trained for two days in customer care and to provide a friendly environment. The pregnant women were also informed of the benefits of attending ANC and delivering at health facilities. 2) Each woman was ***promised*** a mama kit during ANC; 3) ***trust was built*** by showing the mama kit to each woman and branding it with her name; 4) ***keeping the promise*** by providing the mama kit when women came to deliver. The strategy to increase adherence to two doses of SP and encourage women to deliver at health facilities was implemented at two health facilities in Mukono district (Kawolo hospital and Mukono health centre IV). The inclusion criteria were women who: i) consented to the study and ii) were in the second trimester of pregnancy. All pregnant women in the second trimester (4-6 months gestation) who attended ANC and consented to participate in the study were informed of the benefits of SP, the importance of delivering at health facilities, were advised to attend the scheduled visits, promised a mama kit and ensured the kit was available at delivery. The primary outcome was the proportion of pregnant women adhering to a two dose SP regimen.

**Results:**

A total of 2,276 women received the first dose of SP and 1,656 (72.8%) came back for the second dose. 1,069 women were involved in the evaluation (384 had participated in the intervention while 685 had not). The main reasons that enabled those who participated in the intervention to adhere to the two doses of IPTp and deliver at the study facilities were: an explanation provided on the benefits of IPTp and delivering at health facilities (25.1%), availability of a mama kit at delivery (24.6%), kind midwives (19.8%) and fearing complications of pregnancy (8.5%). Overall, 78.0% of these women reported that they were influenced to adhere to IPTp by the intervention. In a multivariable regression, nearby facility, *P* = 0. 007, promising a mama kit, *P* = 0.002, kind midwives, *P* = 0.0001 and husbands’ encouragement, *P* = 0.0001 were the significant factors influencing adherence to IPTp with SP.

**Conclusion:**

The new strategy was a good incentive for women to attend scheduled ANC visits, adhere to IPTp and deliver at the study facilities. Policy implications include the urgent need for developing a motivation package based on the Health-Trust Model to increase access and adherence to IPTp.

## Background

In Uganda, utilisation of health-based interventions like intermittent preventive treatment of malaria in pregnancy (IPTp), antenatal care (ANC) and delivery care is low [1,2]. For example, the proportion of pregnant women who attend the four ANC visits as recommended by the national policy is 47% and those who deliver at health units with skilled attendance is 57%. Similarly, 26.1% of women access two doses of IPTp with SP [2].

Previous studies have assessed factors associated with low utilisation of health-based interventions and documented socio-cultural factors, economic constraints, negative perceptions, poor quality of care and long distances to health units [3-6]. A recent survey in Uganda showed that financial constraints, long distances to the health facilities, concern that drugs were not available and frequent stock-outs of supplies were the most significant factors affecting access to health care [2]. It has been shown that explaining to pregnant women the importance of planning for a delivery led to increased deliveries at health facilities in Uganda [7]. Attending ANC and delivering at health facilities presents an important opportunity for treating complications that arise during delivery and immediately thereafter, and ensuring appropriate referral. Increasing access to essential maternal services in health facilities could dramatically reduce maternal and neonatal morbidity [8-13].

There is now evidence of drug resistance to SP which may have implications for malaria prevention in pregnancy [14-16]. However, SP is still the recommended drug for IPTp in Uganda [17-19]. Thus monitoring patients for resistant parasites during pregnancy is an important strategy to provide data for timely policy review [20]. In order to assess parasite resistance to SP as IPTp in Mukono district, it was important for women to adhere to the recommended two doses of IPTp with SP. Due to poor access to ANC and delivery care in Uganda, an intervention was designed that aimed to increase adherence to a two dose SP regimen and to encourage women to deliver at the facilities. The hypothesis was that a combination of a health messages and the provision of a mama kit would increase adherence to the SP dose regimen and delivery at health facilities. The mama kit was locally assembled composed of a polyethylene sheet (1 metre), four pairs of gloves, cotton wool, gauze, surgical blade, and soap and tetracycline eye ointment.

The aim of the sub-study was to understand more about factors that influence utilisation of health-based interventions like IPTp and delivery care in Uganda in order to improve the design of future interventions and increase access and equity to these services. This article presents results on the evaluation of the new strategy, policy recommendations and areas for further research.

## Methods

### Study area and population

The study was conducted in a malaria endemic district of Mukono in central Uganda. The total population of the district is 850,900 with an annual growth rate of 2.3% and consists predominantly of subsistence farmers of the Baganda ethnic group. The majority of the population, 88%, lives in rural areas. Access to health services is poor and adherence to the two doses of IPTp is currently estimated at 25.9% in the central region where Mukono district is situated. Despite this, ANC attendance for the first visit is high, 94% and women who attend the 4 recommend visits in the central region is 69.1% [2]. Although drugs and supplies in maternity units are supplied free in public facilities as government policy, frequent stock-outs due limited funding and delivery constraints compromise the quality of services.

### Study design

The new strategy was conceived along four constructs of a model (Health-Trust Model) we have constructed, namely: 1) ***creating awareness*** by explaining the benefits of SP and the importance of adhering to the two doses of IPTp. 2) Each pregnant woman attending routine ANC was ***promised*** a mama kit; 3) ***trust was built*** by showing the mama kit to each woman and branding it with her name; 4) ***keeping the promise*** by providing the mama kit when women came to deliver. The mama kit contained 1 metre of a polyethylene sheet, four pairs of gloves, cotton wool, gauze, surgical blade, soap and tetracycline eye ointment. The primary outcome was the proportion of pregnant women who adhered to the two doses of SP. The strategy was evaluated by interviewing women exiting maternity units after delivery from January to December 2011. The evaluation used a quasi-experimental design to assess factors that encouraged women to adhere to IPTp with SP.

The evaluation of this intervention was based on a quasi-experimental study design comparing a sample of women who participated in the intervention and those who did not. Selection of respondents was based on the following criteria: i) any woman who had just delivered at either of the two centres and II) consent to participate in the evaluation. Sample size calculation was based on the proportion of women who completed two doses of SP as IPTp in Uganda estimated at 25% [1]. In order to detect a difference of 6% in this proportion at 80% power and 5% level of significance, a minimum sample of 357 women in each group was required. We aimed to measure the impact of the intervention by asking exiting clients what factors enabled them to adhere to the two doses SP as IPTp and deliver at the health facilities.

The client exit interviews were conducted from January to December 2011. A structured questionnaire was administered to all consenting women exiting the maternity units over a period of 12 month. The client exit questionnaire captured data on demographic characteristics, access to IPTp, delivery experience, client satisfaction, reasons that compelled women to adhere to IPTp and recommendations to attract other women to access essential maternal care.

Twelve midwives working at the two maternity units at Kawolo hospital and Mukono health centre IV conducted the interviews. They were trained for 2 days on study procedures and participated in the pre-testing and revision of the questionnaire before the study. The questionnaire was initially developed in English and translated into the local language (Luganda). The field coordinator and the research team supervised all aspects of data collection.

### Statistical analyses

Data were entered and verified using Microsoft Access 2007 (Microsoft Inc., Redmond, Washington) and analysed using STATA version 11.0 (STATA Corporation, College Station, Texas). Qualitative data was coded and entered. Univariate and bivariate analyses were performed to assess factors that enabled women to adhere to two doses of IPTp with SP, deliver at health facilities and client satisfaction. A binary logistic regression was constructed to analyse factors that enabled women to adhere to IPTp. Variables with a *P-*value less than 0.05 on Univariate analyses were entered into the model using a stepwise procedure. Odds ratios and 95% confidence intervals were calculated. Comparisons between women who participated in the study and those who did not, were made by a chi-square test and a two-sample proportion test. For all calculations, statistical significance was a *P-*value less than 0.05.

### Ethics

Ethical approval for the research was granted from review boards at the Uganda Virus Research Institute and Uganda National Council of Science and Technology (Reference HS. 747). Written consent was obtained from all participating women.

## Results

### Background characteristics

A total of 1,069 women (384 had received two doses of SP as IPTp and delivered at the study facilities; while 685 had not participated in the study but had come to deliver at the study facilities) exiting from maternity units were interviewed. The mean age was 24.1 years, with a range of 14-50 years. The majority of women 63.2% were aged 20-29 years, 43.9% had attained secondary education and 79% were married (Table 1).

**Table 1:** Background characteristics of clients exiting maternity units

**Background characteristics**	**Clients who participated in the study (adhered to IPTp and delivered at study facilities) N = 384**	**Clients who did not participate in the study but delivered at the study facilities N = 685**	**Statistical significance**
**Age (years)**			
14-19	80 (20.8%)	152 (22.2%)	
20-29	233 (60.7%)	433 (63.2%)	
30-50	71 (18.5%)	100 (14.6%)	*X*^2^ = 2.8, *P* =0.25
**Education level**			
None	8 (2.1%)	24 (3.5%)	
Primary	192 (50.0%)	314 (45.8%)	
Secondary	166 (43.2%)	301 (43.9%)	
Tertiary (Technical/University)	18 (4.7%)	46 (6.8%)	*X*^2^ = 9, *P* =0.19
**Marital Status**			
Single	28 (7.3%)	81 (11.8%)	
Married	265 (69.0%)	541 (79.0%)	
Cohabiting	80 (20.8%)	48 (7.0%)	
Widowed/Separated	11 (2.9%)	15 (2.2%)	*X*^2^ = 52, *P =* 0.000

### Adherence to IPTp

The intervention recruited 2,276 women on the first dose of SP; and of these 1,656 (72.8%) came back for the second dose of SP while 937 (41.2%) came to deliver at the study facilities (Table 2). Women exiting maternity units were asked which factors led them to adhere to the two doses of IPTp with SP and deliver at the health facilities. A quarter of the women, 25.1% mentioned the advice given during ANC on the benefits of IPTp and delivering at health facilities; 24.6% mentioned the promise of the mama kit; 19.6% mentioned that it was because the health facility was near to their homes; and 8.5% feared the complications of pregnancy.

**Table 2:** Adherence to IPTp at the study facilities

**Recruitment of women**	**Number of women recruited**	**Percentage**
Number of pregnant women receiving the first dose of SP (N)	2,276	-
Number of women receiving the second dose of SP (N_1_)	1,656	72.8%+
Number of women delivering at study facilities N_2_)	937	41.2%*

+Proportion of women adhering to two doses of SP, N_1_/N*100 = 72.8%.

*Proportion of women adhering to the advice to delivery at health facilities, N_2_/N*100 = 41.2%.

### Client satisfaction

Most deliveries were assisted by midwives; and an overwhelming majority were satisfied with the care they had received. Majority of women said they would deliver their next babies at health facilities. Similarly, the majority of women, 86.2%, said they were impressed by the explanation on the benefits of IPTp, the promise of the mama kit and the attention they received at the maternity units. A small proportion (7.9%) said they were pleased to find out their HIV status and the presence/absence of malaria infection (Table 3).

**Table 3:** Clients’ experiences at the study facilities

**Clients’ experiences**	**Clients who participated in the study (adhered to IPTp and delivered at study facilities) N = 384**	**Clients who did not participate in the study but delivered at the study facilities N = 685**	**Statistical significance**
**Who assisted you to deliver the baby?**			
Doctor	39 (10.2%)	51 (7.5%)	
Midwife	324 (84.4%)	569 (83.1%)	
Nursing aide	11 (2.9%)	55 (8.0%)	
Self	10 (2.5%)	10 (1.5%)	*X*^2^ = 17, *P* =0.002
**Were you satisfied with the help?**			
Yes	378 (97.8%)	670 (97.8%)	
No	6 (2.2%)	15 (2.2%)	*X*^2^ = 0.5, *P* =0.5
**How do you rate your health today?**			
Very Good	47 (12.2%)	131 (19.1%)	
Good	264 (68.8%)	492 (71.8%)	
Fair	71 (18.5%)	60 (8.8%)	
Poor	2 (0.5%)	2 (0.3%)	*X*^2^ = 29, *P* =0.000
Will you deliver your next baby at this facility?*			
Yes	362 (97.8%)	620 (98.9%)	
No	8 (2.2%)	7 (1.1%)	*X*^2^ = 3, *P* =0.25
**Person accompanying pregnant woman**			
Husband	108 (28.1%)	239 (34.9%)	
Mother	73 (19.0%)	173 (25.3%)	
Sister	91 (23.7%)	143 (20.9%)	
Nobody	112 (29.2%)	130 (19.0%)	
**What did you like in this study**			
Received an explanation on the benefits of preventing malaria in pregnancy and was given a mama kit and I received immediate attention at delivery	175 (86.2%)	-	
Immediate care and attention given’ during ANC	22 (10.8%)	-	
Was asked questions in a friendly way	3 (1.4%)	-	
Everything done to me was good	1 (0.5%)	-	
I found out my HIV status and whether infected with malaria	16 (7.9%)	-	
Received advice and treatment of other diseases	2 (1.0%)	-	
Discovered what happens in a health facility	2 (1.0%)	-	
Got information on family planning	4 (2.0%)	-	

*Missing data.

As seen from Table 4, the intervention benefited other women who did not participate in the study since they attended the same ANC clinics or they could have benefited from other incentives at health facilities (Table 4).

**Table 4:** Explanatory factors to adherence to IPTp

**Re Factors, reasons and recommendations**	**Clients who participated in the study (adhered to IPTp and delivered at study facilities)**	**Clients who did not participate in the study but delivered at the study facilities**	**Statistical significance**
**What important factor enabled you to complete two doses of SP and come to deliver at this facility?**			
Was given an explanation on benefits of SP and delivering at health facilities for me and the baby	95 (25.1%)	108 (16.1%)	*X*^2^ = 30, *P* =0.000
Was promised a mama kit and it reduced costs	93 (24.6%)	124 (18.4%)	
Facility is near my home	74 (19.6%)	153 (22.7%)	
Midwives were kind to me during ANC and at delivery	75 (19.8%)	183 (27.2%)	
I feared complications during delivery	32 (8.5%)	61 (9.1%)	
My husband encouraged me to attend ANC and to come to deliver	9 (2.4%)	44 (6.5%)	
**Reasons given by women who participated in the study as to why other women don’t access maternity care at health facilities**			
Long distances to health facilities	124 (32.6%)	193 (28.6%)	
Women don’t know complications during labour and delivery	18 (4.7%)	47 (7.0%)	
The midwives are not friendly	17 (4.5%)	58 (8.6%)	
High costs involved	115 (30.1%)	202 (30.0%)	
Husbands don’t provide support	69 (18.1%)	135 (20.0%)	
Lack of drugs	38 (10.0%)	39 (5.8%)	*X*^2^ = 17, *P* =0.001
**What do you recommend to encourage women to access maternity care at health facilities?**			
To provide mama kits, drugs, and gloves at delivery	83 (24.5%)	141 (26.1%)	
To provide information on complications of pregnancy and labour	21 (6.2%)	45 (8.3%)	
Education on benefits of ANC and delivering at health facilities	80 (23.6%)	79 (14.6%)	
Construct nearby health facilities	66 (19.5%)	106 (19.6%)	
Involve men in ANC	5 (1.5%)	29 (5.4%)	
Health workers need to be friendly	9 (2.7%)	20 (3.7%)	
Employ more health workers	37 (10.9%)	73 (13.5%)	
Provide ambulances	28 (8.3%)	21 (3.9%)	
Renovate the maternity units	10 (2.9%)	26 (4.8%)	*X*^2^ = 36, *P* =0.009

The factors that most influenced women to adhere to the two doses of IPTp with SP and deliver at the facilities were: the promise of a mama kit (*P* = 0.002), the facility being nearby (*P* = 0.007), husbands encouraging their spouses (*P* = 0.0001), midwives being kind (*P* = 0.0001), and the education level of the woman (*P* = 0.025), (Table 5).

**Table 5:** Factors related to access and adherence to IPTp

**Covariate**	**Odds ratio**	**95% CI**	**Statistical significance**
Education level	0.875	0.768 - 0 .996	0.025
Marital status	1.017	0.975 - 1.061	0.426
Age	1.016	0.992 - 1.041	0.189
Nearby health facility	0 .573	0.383 - 0 .859	0 .007
Promised a mama kit	0.586	0.322 - 1.065	0.002
Midwives were kind to me	0.480	0.323 - 0 .713	0.0001
My husband encouraged me	0.246	0.113 - 0 .533	0.0001

## Discussion

The new strategy influenced pregnant women to complete two doses of SP. The main elements of the intervention that enabled women to adhere to the two doses of IPTp and deliver at the study facilities were: training midwives to explain the benefits of IPTp and delivering at health facilities for the mother and the baby and to provide good customer care by greetings all patients and creating a friendly environment. Midwives also ensured that a mama kit was available at delivery. The promise of a mama kit seems to have motivated women to attend the required numbers of ANC and come to deliver at the facilities. This is because availing the mama kit offsets substantial costs to women of approximately US $5. We recommend studies that evaluate other incentive schemes to enable pregnant women access essential maternal care at both public and private sectors. Such studies would contribute to the current effort to develop and implement a health insurance policy in Uganda.

The midwives who implemented the intervention were motivated by a small allowance of US $25 per month and regular supervision from the research team. There is need to identify and cost several incentive schemes in order develop a motivation package for service providers in order to improve quality of services.

Other women mentioned a mama kit as one of the factors that enabled them to deliver at the health facilities. This is because the Ministry of Health in Uganda has prioritised health supplies including a mama kit [1]. However, one of the constraints is frequent stock-outs. In this study, we ensured that all pregnant women who consented to participate in the study were promised and received a mama kit at delivery. Despite this, relatively few women delivered at health facilities (Table 2). Constraints like high costs, long distances to health facilities and the role of the husband could have influenced this outcome (Table 4).

It is worth noting that the role of the husband is important for pregnant women to access IPTP and delivery care at health facilities. Of equal importance is the attitude and conduct of midwives towards pregnant women. Thus, efforts should target spouses and support midwives to provide good customer care.

A significant proportion of women said they were pleased to know their HIV status and whether they were infected with malaria at the time of delivery. This is an important finding since women are supposed to access routine HIV counselling and testing during ANC. During the study, the importance of knowing their HIV status was discussed with participating women. Due to staffing problems, many women attending ANC do not get this opportunity and the recommendation to recruit more midwives is in line with this finding. Similarly, the introduction of rapid diagnostic tests for malaria at health facilities would provide women with access to testing and treatment during pregnancy.

In interpreting the findings of the present study, there is need to note that the intervention was not randomised and therefore there are many other factors that could have influenced women to adhere to IPTp and deliver at the health facilities. For example, socio-economic factors were not fully explored such as household income and expenditure patterns which have an influence on health seeking practices. It is important to continue exploring this subject further since health seeking behaviour is influenced by several factors that change with time.

Previous studies [21,22] have proposed explanatory factors that influence individuals to access health services and adhere to the recommendations for positive behavioural practices. Based on the present results which show that actions at health facilities that promise clients a benefit for using health interventions and builds trust between the health provider and the clients; we propose a conceptual framework that summarises these constructs into a “Health-Trust Model” (HTM). As an area for further research, we propose that the robustness of the model be tested in order to understand fully how it explains access and adherence to IPTp and other health interventions.

The implications of these results are that the health system in Uganda needs to put the midwife at the forefront in order to have positive maternal outcomes. This implies recruiting more midwives in both the ANC and maternity units. The midwives need to be facilitated and mentored to provide good quality of care. For the sustainability of this intervention, there is a need to design a good logistical supply chain to ensure no stock-outs of essential commodities especially mama kits. The government needs to construct more health facilities with maternity units in order to reduce the long distances that pregnant women have to walk to access a health facility. Currently there are no delivery facilities at health centres II which are based at the parish level. This is a geographical area serving a population of 5,000 people. This should be a priority option to increase the number of health facilities offering IPTp and maternity services at this level.

## Conclusion

The new strategy was a good incentive for women to attend scheduled ANC visits, adhere to IPTp and deliver at the study facilities. Policy implications include the urgent needfor developing a motivation package based on the Health-Trust Model to increase access and adherence to IPTp. The robustness of the Health Trust Model (HTM) could be tested further to explain access to essential maternity care as well as access to other health interventions like insecticide treated nets (ITNs).

## Competing interests

The authors declare that they have no competing interests.

## Authors’ contributions

AKM designed the study, analysed data and wrote the manuscript; SY and JB participated in study implementation and reviewed the manuscript; and PM participated in the study design, oversight supervision and reviewed the manuscript. All authors read and approved the final manuscript.

## Pre-publication history

The pre-publication history for this paper can be accessed here:

http://www.biomedcentral.com/1471-2393/13/178/prepub
